# Habitat Analysis for Risk Prediction of Nasopharyngeal Carcinoma: A Comparative Study of Different MRI Sequences and Regional Combinations

**DOI:** 10.3390/bioengineering13050521

**Published:** 2026-04-29

**Authors:** Zijun Huang, Yu Li, Jia Kou, Shanqi Bao, Ying Sun, Li Lin

**Affiliations:** 1 School of Biomedical Engineering, Southern Medical University, No. 1023-1063, Shatai South Road, Baiyun District, Guangzhou 510515, China; hzj286534644@gmail.com (Z.H.); liyu18@sysucc.org.cn (Y.L.); sqbao@smu.edu.cn (S.B.); 2 North Campus, Sun Yat-sen University, No. 74, 2nd Zhongshan Road, Yuexiu District, Guangzhou 510080, China; koujia@sysucc.org.cn; 3 Department of Radiation Oncology, Sun Yat-sen University Cancer Center, No. 651 Dongfeng East Road, Yuexiu District, Guangzhou 510060, China; 4 Department of Clinical Research, State Key Laboratory of Oncology in South China, Guangdong Key Laboratory of Nasopharyngeal Carcinoma Diagnosis and Therapy, Sun Yat-sen University Cancer Center, No. 651 Dongfeng East Road, Yuexiu District, Guangzhou 510060, China

**Keywords:** nasopharyngeal carcinoma, magnetic resonance imaging, habitat analysis, multi-regional spatial interaction, GTVp-MLN region combinations, risk prediction

## Abstract

Habitat analysis enables spatial characterization of intratumoral heterogeneity; however, its application in nasopharyngeal carcinoma (NPC), particularly regarding metastatic lymph node (MLN), remains limited. This study aims to systematically compare the prognostic performance of various models using different sequences and spatial region combinations for predicting overall survival in NPC. The study retrospectively included 725 NPC patients (543 training, 182 testing). Habitat analysis was conducted based on T1, T1C, and T2 sequences in three regional strategies: primary gross tumor volume (GTVp), metastatic lymph nodes (MLNs), and the combined region of GTVp-MLN. The tumor area was divided into six subregions, and a multi-region spatial interaction (MSI) matrix was constructed to extract MSI features. On this basis, a radiomics model (R Model) and a clinical–radiomics model (CR Model) were established, and the model performance was evaluated using C-index and Kaplan–Meier survival analysis. The results show that the combined GTVp-MLN model based on the T1 sequence achieved the best overall predictive performance (R Model: C-index = 0.693; CR Model: C-index = 0.722). Significant survival differences were observed between the high- and low-risk groups. These findings suggest that habitat analysis incorporating the combined GTVp–MLN region may improve prognostic prediction and risk stratification in patients with NPC.

## 1. Introduction

Nasopharyngeal carcinoma (NPC) is a malignant tumor of the head and neck characterized by distinct geographic distribution. Although the global annual incidence is less than 1 per 100,000, NPC is highly prevalent in southern China, where approximately 50% of newly diagnosed cases and deaths worldwide occur each year [1,2]. With the development of intensity-modulated radiotherapy (IMRT) and multimodal treatment strategies, the overall survival (OS) of NPC patients has improved substantially. However, a proportion of patients still develop distant metastasis or recurrence, indicating that the biological behavior of NPC exhibits significant spatial heterogeneity [3,4].

Magnetic resonance imaging (MRI), including T1-weighted (T1), T2-weighted (T2), and contrast-enhanced T1-weighted (T1C), can characterize tumor features from multiple dimensions such as tissue structure, water content, and vascular supply, and it has become a core imaging modality for the diagnosis, staging, and treatment evaluation of NPC [5]. Conventional radiomics typically assumes that tumor heterogeneity is well mixed within the tumor region and extracts averaged quantitative features from the entire tumor volume [6,7]. However, this approach overlooks the spatial heterogeneity present within tumors. O’Connor et al. emphasized that characterizing intratumoral subregional structures is crucial for understanding tumor biological behavior [8]. Habitat analysis provides a novel strategy for identifying tumor microenvironments. Based on traditional radiomics, this method clusters intratumoral imaging phenotypes and partitions tumors into multiple subregions with similar biological characteristics, thereby enabling spatial characterization of tumor heterogeneity and revealing the spatial distribution of distinct biological properties within the tumor microenvironment [9]. Wu et al. proposed a multi-region spatial interaction (MSI) matrix for risk stratification, achieving superior predictive performance compared with conventional imaging biomarkers in breast cancer [10]. Similarly, Xue et al. developed an interpretable habitat analysis model based on PET imaging that accurately predicted progression-free survival in patients with early-stage non-small cell lung cancer, significantly improving risk stratification [11]. These studies suggest that habitat analysis may help uncover spatial heterogeneity biomarkers with potential biological significance.

However, systematic studies of habitat analysis in NPC remain limited. Most existing radiomics prediction models are primarily constructed based on the primary gross tumor volume (GTVp), with insufficient utilization of spatial information from metastatic lymph node (MLN) regions. Yuan et al. developed a habitat analysis model based on intratumoral heterogeneity within GTVp and demonstrated promising predictive performance in locally advanced NPC [12]. Nevertheless, their study did not incorporate spatial heterogeneity information from MLN. In fact, MLN status is closely associated with the risk of distant metastasis and recurrence in NPC [13]. Whether MLN should be incorporated into the habitat analysis framework and whether joint modeling of GTVp and MLN can significantly improve predictive performance remain unclear. In addition, different MRI sequences exhibit varying sensitivities to tissue composition and vascular characteristics, and their influence on survival prediction within the habitat analysis framework has not yet been systematically investigated. Consequently, methodological gaps remain regarding the optimal sequence selection and regional combination strategies for habitat analysis modeling.

To address these gaps, this study was designed with the following objectives:

- To compare the prognostic performance of habitat features derived from T1, T1C, and T2 MRI sequences for OS prediction in NPC.
- To evaluate the prognostic significance of habitat subregional information derived from MLN.
- To determine the optimal regional strategy for habitat-based risk stratification among GTVp, MLN, and the combined GTVp-MLN region.

Guided by these objectives, this study systematically evaluated the impact of different MRI sequences and the combination strategies of GTVp and MLN regions on the performance of prognostic models based on habitat analysis for predicting OS in NPC patients, while further investigating whether the integration of clinical variables with MSI features could enhance predictive performance.

The remainder of this paper is organized as follows. Section 2 introduces the study materials and methodological framework, including habitat analysis, MSI feature extraction, and risk stratification. Section 3 presents the comparative results of risk classification and prognostic modeling across different MRI sequences and regional strategies. Section 4 discusses the major findings, clinical implications, limitations, and future research directions.

## 2. Materials and Methods

### 2.1. Study Population

This retrospective study included 725 pathologically confirmed non-metastatic NPC patients from XX Cancer Center. All patients underwent multi-sequence MRI scans (T1, T1C, and T2 sequences) before induction chemotherapy (IC) between 1 February 2010 and 1 April 2016. The cohort was randomly divided into a training set (n = 543) and a testing set (n = 182) at a ratio of 3:1. The inclusion criteria were as follows: (1) pathologically confirmed non-metastatic NPC; (2) TNM stage III–IVA disease; (3) MRI examination of the nasopharynx and neck performed within four weeks before IC; (4) quantitative plasma cell-free Epstein-Barr virus (cfEBV) DNA measurement performed before IC. The exclusion criteria included: (1) absence of MLN segmentation masks before IC; (2) incomplete clinical data; (3) poor MRI image quality. Detailed treatment strategies are provided in the Appendix A.

### 2.2. Study Endpoints and Patient Follow-Up

The clinical endpoint of this study was OS, defined as the time from the initiation of treatment to death from any cause. The follow-up period was calculated from the date when treatment began until the last follow-up visit or death. Patients were followed every 3 months for the first 3 years after IC, every 6 months during 4–5 years, and annually thereafter until death or loss to follow-up. Follow-up assessments included nasopharyngeal endoscopy, plasma cfEBV DNA testing, MRI from the suprasellar cistern to the clavicular region, CT scans, abdominal ultrasound, whole-body bone scintigraphy (ECT), and 18F-FDG PET-CT when indicated. Suspected recurrent or metastatic lesions were confirmed by histopathological examination.

### 2.3. MRI Acquisition and ROI Delineation

MRI data were obtained from the Picture Archiving and Communication System (PACS) and used for region of interest (ROI) delineation and analysis. The GTVp and MLN were initially segmented on each T1C and T2 sequence using an automatic contouring system. All segmentations were subsequently reviewed and, when necessary, corrected by an experienced radiation oncologist, with additional manual adjustments performed as required. To ensure the consistency of ROIs across different MRI sequences, T1 and T2 images were registered to the T1C sequence using the Greedy Registration toolkit implemented in Python 3.7 [14]. Detailed MRI acquisition and preprocessing protocols are provided in the Appendix B.

### 2.4. Study Workflow

**Figure 1:**
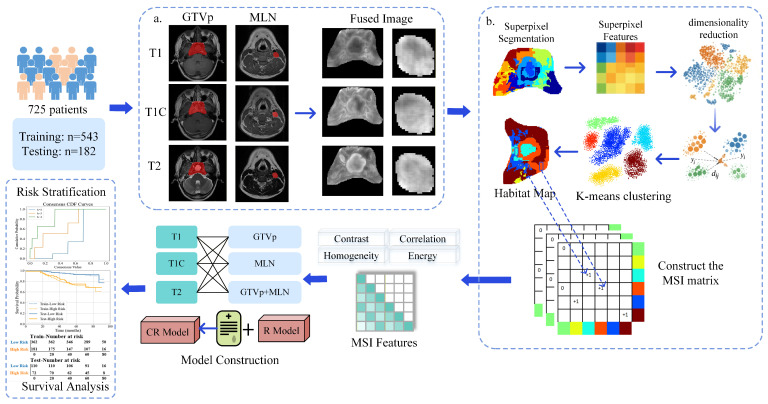
The workflow of study: (**a**) Image preprocessing, the red regions indicate tumor regions of interest (ROIs), including GTVp and MLN; (**b**) Habitat analysis, different colors in the habitat maps and clustering diagrams represent different habitat subregions or clusters.

The workflow of this study is illustrated in Figure 1. First, MRI images were standardized and preprocessed to generate fused images. Subsequently, habitat analysis was performed on the fused images. Through segmentation, dimensionality reduction, and clustering, the tumor region was divided into six habitat subregions with similar imaging characteristics, generating a habitat map. Based on these habitat subregions, a MSI matrix was constructed to extract MSI features. MSI features were then extracted from T1, T1C, and T2 sequences within the GTVp and MLN regions, and further combined to form GTVp-MLN features. Consensus clustering was subsequently applied to determine risk categories. Radiomics models (R Model) were constructed based on MSI features, and clinical variables were further incorporated to develop clinical–radiomics models (CR Model). Finally, model performance in predicting OS was evaluated and compared using the concordance index (C-index) and Kaplan-Meier survival analysis.

### 2.5. MRI Image Preprocessing

**Figure 2:**
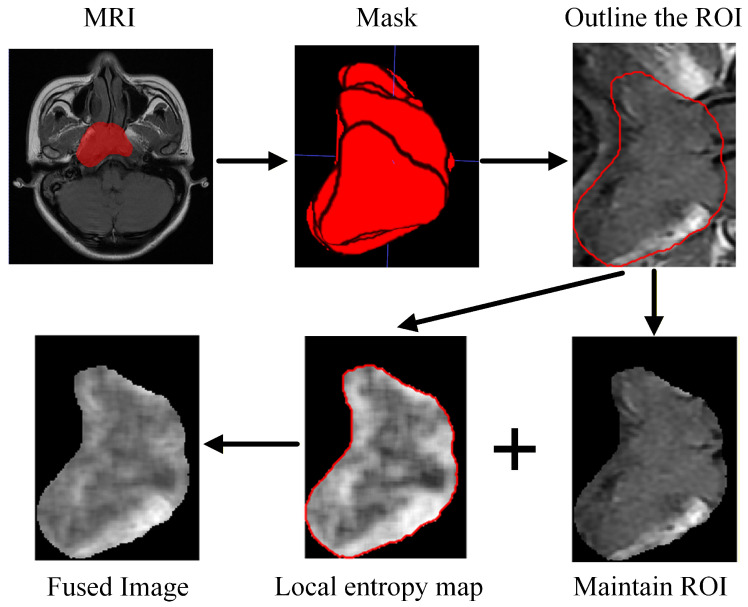
Image preprocessing flow, arrows indicate the sequential steps, the red regions indicate tumor ROIs.

Before habitat analysis, standardized preprocessing was performed on the original MRI images to reduce the influence of different scanning conditions and noise on image analysis and to improve the stability of subsequent habitat construction (Figure 2). The preprocessing procedures mainly included intensity normalization, ROI cropping, boundary smoothing, and texture enhancement.

First, intensity normalization was applied to MRI images of each sequence for every patient to reduce grayscale variations caused by different scanners and acquisition parameters, thereby improving data consistency across patients. Subsequently, the corresponding tumor masks were loaded, and the morphologically eroded masks were automatically cropped to retain only the ROI, which reduced background information and improved computational efficiency.

To enhance local texture information within the images, a local entropy map was further calculated from the normalized MRI images to characterize the complexity of grayscale distribution within pixel neighborhoods. The local entropy map was then voxel-wise added to the original ROI image to generate an entropy-enhanced fused image. In other words, each voxel intensity in the fused image was obtained by adding the original ROI intensity to its corresponding local entropy value [15]. This fusion strategy preserved structural intensity information while improving the representation of local texture contrast and spatial heterogeneity [16]. The fused image was subsequently used as the input for superpixel segmentation, habitat construction, and MSI feature extraction, which helped improve the accuracy and stability of habitat subregion delineation.

### 2.6. Habitat Analysis

The workflow of habitat analysis is illustrated in Figure 1b, which mainly includes individual level superpixel segmentation, population level clustering analysis, and construction of the MSI matrix.

At the individual level, to characterize the spatial heterogeneity within tumors, the Simple Linear Iterative Clustering (SLIC) algorithm was first applied to the fused image for superpixel segmentation [17]. In this study, the number of superpixels was adaptively determined according to the ROI volume, ranging from 30 to 100, while the compactness parameter was empirically set to $1\times 10^{-2}$ to balance local texture preservation and spatial continuity in MRI-based habitat partitioning. This approach divides the tumor region into multiple spatially contiguous superpixels with relatively homogeneous imaging phenotypes. SLIC maps voxels into a feature space composed of intensity values and spatial coordinates, and iteratively clusters voxels with similar intensity, texture, and spatial proximity into compact regions with continuous boundaries. This process effectively reduces data dimensionality while preserving local structural information [18,19]. Subsequently, intensity, texture, and statistical features were extracted from each superpixel region to construct superpixel feature vectors that describe the imaging phenotype patterns within the tumor.

At the population level, to identify potential spatial heterogeneity patterns across different patients, the superpixel features from all patients were first aggregated to form a high-dimensional feature space. The t-distributed stochastic neighbor embedding (t-SNE) algorithm was then applied for nonlinear dimensionality reduction to preserve local neighborhood relationships and facilitate robust clustering [20,21]. The Barnes-Hut implementation with MATLAB 2021 default settings was adopted, including a perplexity of 30 and a maximum of 1000 iterations. In the reduced feature space, the K-means algorithm [22] was further used for clustering, and the clustering labels were mapped back to the original image space to generate habitat maps with clear spatial structures. The maximum number of iterations was set to 100, with 10 replicates to improve clustering robustness, while the default k-means++ centroid initialization strategy was used.

Based on the resulting habitat maps, a MSI matrix was constructed to quantify the spatial adjacency relationships between different habitat subregions as well as within each subregion. This matrix characterizes the spatial organizational patterns of the tumor microenvironment and provides stable and biologically interpretable features for subsequent survival prediction models.

### 2.7. MSI Features Extraction

After constructing the MSI matrix, the spatial adjacency relationships among different habitat subregions were statistically quantified. In this way, the complex spatial organization within the tumor was transformed into a regular matrix representation, enabling quantitative characterization of tumor spatial interaction patterns.

First, texture modeling was performed on the MSI matrix using the gray-level co-occurrence matrix (GLCM) method. Typical texture features, including contrast, homogeneity, correlation, and energy, were extracted to characterize differences in spatial interaction patterns and the structural stability among different habitat subregions [23].

To further distinguish between the heterogeneity between subregions, the diagonal elements of the MSI matrix were separately extracted. The diagonal elements represent the spatial interaction relationships among pixels within the same habitat subregion, thereby reflecting the internal structural consistency and stability of each subregion. In contrast, the lower triangular elements characterize the spatial adjacency and interaction patterns between different habitat subregions, reflecting the spatial heterogeneity and potential cooperative evolution among different functional tumor subregions.

MSI features were extracted separately from the GTVp and MLN regions. The extracted features were then concatenated to construct a combined GTVp-MLN feature set, which enhances the overall representation ability while preserving region-specific information. All features were batch-extracted using MATLAB, resulting in a habitat imaging feature set for subsequent model construction (Table 1).

### 2.8. Risk Classification Based on MSI Features

To explore the clustering performance of MSI features for risk stratification, an unsupervised clustering analysis based on MSI features was first performed. A similarity matrix among patients was constructed using the Spearman rank correlation coefficient calculated from the MSI feature profiles. Based on this matrix, the k-medoids clustering algorithm was applied to partition patients into groups, with the number of clusters set to *k* = 2–5 to explore the optimal clustering solution.

To improve the stability and robustness of the clustering results, a consensus clustering strategy was adopted [24]. Through repeated subsampling and multiple clustering iterations, clustering performance under different cluster numbers was evaluated using internal clustering validation metrics, including the Silhouette coefficient [25] and the Calinski–Harabasz (CH) score [26]. A Silhouette coefficient closer to 1 indicates better clustering quality, while a higher CH score suggests more compact clusters and greater separation between clusters. Based on the comprehensive evaluation of these metrics, the optimal number of clusters was determined. Finally, patients were categorized into high and low risk groups according to the optimal clustering results for subsequent survival analysis.

Furthermore, based on previously reported clinical factors with significant prognostic value, including age, T stage, N stage, and pre-IC plasma cfEBV DNA level [27], three prognostic models were constructed: Clinical model (C Model) based solely on clinical variables; R Model based on the predefined 58 MSI features derived from the six-subregion habitat interaction framework; and CR Model integrating both clinical variables and MSI features. Because the MSI features were generated from a biologically structured habitat topology rather than unrestricted high-dimensional radiomic extraction, explicit feature selection was not additionally performed. Instead, a penalized Cox proportional hazards model with L2 regularization was adopted to improve model robustness, reduce the risk of overfitting, and stabilize coefficient estimation [28].

The predictive performances of these models were compared to evaluate the incremental value of multimodal information in predicting OS in NPC patients.

### 2.9. Statistical Analysis

According to previous studies, pre-IC plasma cfEBV DNA levels were categorized into two groups using a cutoff value of <4000 copies/mL and ≥4000 copies/mL [29]. Age was dichotomized based on the median value of the entire cohort. Differences in the distribution of clinical variables between groups were compared using the Pearson’s $\chi^{2}$ test or Fisher’s exact test. The predictive performance of the models was evaluated using C-index and the area under the receiver operating characteristic (ROC) curve (AUC) [30]. Both metrics range from 0 to 1, with values closer to 1 indicating stronger predictive ability.

Based on the previously determined consensus clustering results, patients were categorized into different risk groups for survival analysis. Kaplan–Meier survival curves were plotted to estimate survival probabilities, and differences between risk groups were compared using the log-rank test [31].

All statistical tests were two-sided, and a p<0.05 was considered statistically significant. All statistical analyses were performed using Python (version 3.9), primarily with the lifelines, scikit-survival, and scikit-learn packages.

## 3. Results

### 3.1. Patient Characteristics

Table 2 summarizes the baseline clinical characteristics of patients in the training and testing sets. A total of 543 patients were included in the training set, including 415 males and 128 females, with a median age of 43 years. The testing set consisted of 182 patients, including 137 males and 45 females, with the same median age of 43 years. The median follow-up times were 64.9 months for the training set and 66.7 months for the testing set. By the end of follow-up, 82 patients (15.1%) in the training set experienced study endpoint events (death, recurrence, or distant metastasis), while 28 patients (15.3%) in the testing set experienced endpoint events. Statistical analysis showed no significant differences in baseline clinical characteristics between the training and testing set (p>0.05), indicating good balance between the two datasets and supporting their suitability for subsequent model development and validation.

### 3.2. Quantitative Determination of the Optimal Number of Habitat Subregions

To quantitatively determine an appropriate number of habitat subregions, three candidate clustering schemes (k=4, k=6, and k=8) were systematically compared. The same internal clustering validation metrics used for patient risk classification, namely the Silhouette coefficient and CH score, were further applied here to quantitatively evaluate the clustering quality of habitat subregion partitioning.

**Figure 3:**
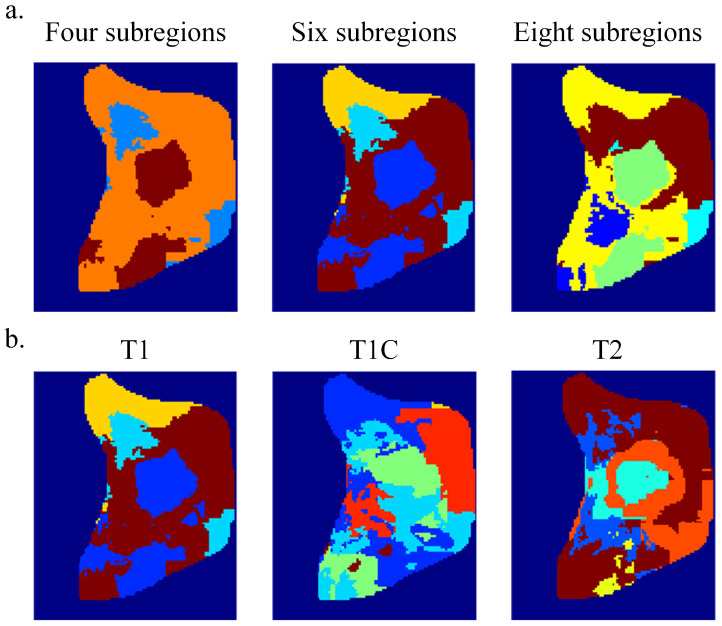
Habitat imaging results: (**a**) Comparison of habitat partitioning under different clustering numbers (k=4,6, and 8). (**b**) Representative habitat maps derived from T1, T1C, and T2 MRI sequences using the optimal clustering number (k=6). Different colors represent distinct habitat subregions identified by clustering.

As illustrated in Figure 3a, increasing the number of habitat subregions led to progressively finer spatial partitioning of the tumor, allowing more detailed depiction of intratumoral heterogeneity patterns. However, excessive subdivision may also introduce fragmented regions and reduce the stability of habitat representation.

To further support the selection quantitatively, the clustering performance of the three candidate schemes was evaluated using the Silhouette coefficient, CH score, and the resulting MSI feature dimensionality (Table 3). Among them, k=6 achieved the highest Silhouette coefficient and CH score, indicating the best balance between intra-cluster compactness and inter-cluster separability. Although k=8 provided finer habitat partitioning, it substantially increased MSI feature dimensionality from 58 to 92 features, which may introduce unnecessary model complexity and increase the risk of overfitting in subsequent prognostic modeling. In contrast, k=4 produced lower clustering quality and a relatively coarse spatial characterization. Therefore, considering both visual interpretability and quantitative clustering performance, k=6 was selected as the optimal number of habitat subregions for subsequent analysis.

Using the optimal clustering number (k=6), representative tumor habitat maps constructed from different MRI sequences are shown in Figure 3b. It can be observed that different sequences exhibit certain variations in depicting the internal spatial structure of tumors. The T1, T1C, and T2 sequences present distinct signal distribution patterns in the fused images, and accordingly, their habitat partitions demonstrate different spatial morphologies and regional distributions.

Overall, the habitat maps effectively reflect the spatial heterogeneity within tumors. Different habitat subregions exhibit relatively continuous spatial distributions with clear boundaries, suggesting that this method can effectively identify potential imaging phenotype differences within tumors. This provides a robust and biologically meaningful basis for subsequent MSI feature extraction and prognostic risk stratification.

### 3.3. Optimization of Risk Stratification Based on MSI Features

To optimize risk stratification based on MSI features, unsupervised clustering was performed on the extracted MSI features to divide patients into different risk groups. The clustering performance under different cluster numbers (*k* = 2–4) was evaluated using the Silhouette coefficient and the CH score, which measure cluster compactness and inter-cluster separation.

**Figure 4:**
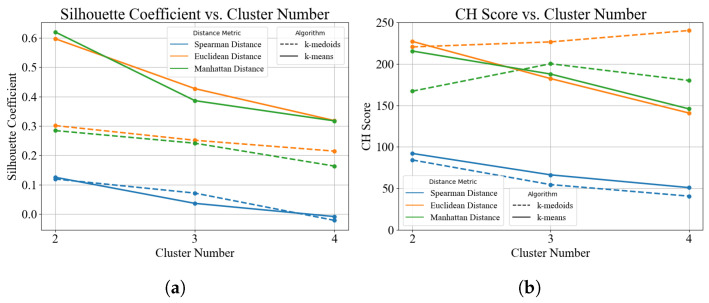
Clustering performance evaluation for determining the optimal risk groups (*k* = 2–4): (**a**) Silhouette coefficient across different cluster numbers. (**b**) CH score across different clustering strategies and distance metrics. The distance measurement and clustering methods are distinguished by different line types and colors.

As shown in Figure 4, when the number of clusters was set to 2, most clustering strategies achieved higher Silhouette coefficients and CH scores, indicating more compact clusters and greater separation between groups. With increasing cluster numbers, the Silhouette coefficient showed a decreasing trend, suggesting that further subdivision did not improve the clustering structure and might reduce clustering stability.

Sensitivity analyses using different clustering algorithms (k-medoids and k-means) and distance metrics (Spearman, Euclidean, and Manhattan distances) produced consistent results. Overall, *k* = 2 demonstrated the best clustering stability and separation performance. Therefore, *k* = 2 was selected as the optimal clustering solution, and patients were finally stratified into two risk groups: low risk group and high risk group.

### 3.4. Prognostic Model Construction and Evaluation

To systematically evaluate the prognostic performance of different MRI sequences and tumor region combinations, R Model were constructed based on MSI features extracted from T1, T1C, and T2 sequences within the GTVp and MLN regions. Furthermore, clinical variables were integrated to develop CR Model, with OS as the study endpoint.

The predictive performance of different models is summarized in Table 4 and Table 5. Overall, models constructed based on the combined GTVp–MLN region demonstrated better predictive performance than those based on single regions in both the training and testing cohorts. This finding suggests that analyzing either the GTVp or MLN region alone may not fully capture the spatial heterogeneity of NPC, whereas integrating information from both regions provides a more comprehensive characterization of tumor microenvironment complexity.

When comparing different MRI sequences, models based on the T1 sequence showed relatively stable and superior predictive performance. As shown in Table 4, the R Model based on GTVp-MLN combination of T1 sequence achieved a C-index of 0.713 in the training set and 0.693 in the testing set, with corresponding AUC values of 0.724 and 0.712, respectively. In contrast, models based on T1C and T2 sequences generally showed slightly lower predictive performance or reduced stability across datasets.

After incorporating clinical variables, model performance was further improved. As shown in Table 5, the CR Model outperformed the R Model in most sequence and region combinations. Among them, the CR Model based on GTVp-MLN combination of T1 sequence achieved the best predictive performance, with a C-index of 0.735 in the training set and 0.722 in the testing set, and corresponding AUC values of 0.756 and 0.742, respectively. For reference, the C Model achieved training C-index of 0.591 and testing C-index of 0.525, which were lower than that of the CR Models, further supporting the added prognostic value of MSI features.

Overall, these results indicate that integrating MSI features with clinical variables significantly improves prognostic prediction performance. The CR Model based on GTVp-MLN combination of T1 sequence was therefore identified as the optimal predictive model in this study and was subsequently used for further survival analysis.

### 3.5. Survival Analysis Based on the GTVp–MLN Combination Prognostic Model

To further evaluate the clinical utility of the optimal prognostic model for patient risk stratification, patients were categorized into high and low risk groups according to the risk clusters derived from the consensus clustering analysis. Kaplan–Meier survival curves were subsequently plotted using OS as the clinical endpoint.

**Figure 5:**
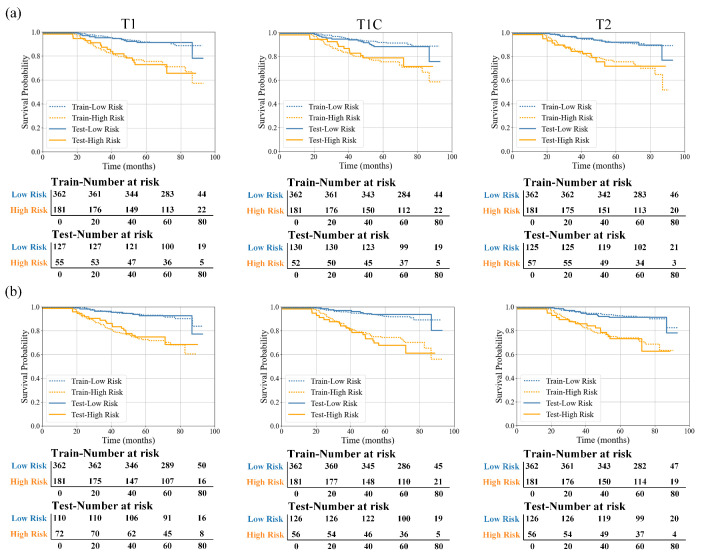
Kaplan–Meier survival curves for survival stratification using the GTVp–MLN combination prognostic model across different MRI sequences (T1, T1C, and T2): (**a**) R Model; (**b**) CR Model.

As shown in Figure 5, the high risk group consistently exhibited significantly lower OS compared with the low risk group across different MRI sequences. Similar survival separation trends were observed in both the training and testing sets. Among the evaluated models, the CR Model based on the combined GTVp–MLN region demonstrated clear separation of survival curves across all MRI sequences, with log-rank tests indicating significant survival differences between the two groups (*p* < 0.05).

In comparison, the R Model, which was constructed solely from MSI features, was also able to distinguish patients with different prognoses to a certain extent, but the separation between survival curves was relatively weaker. Overall, the CR Model integrating clinical variables showed more stable and pronounced survival discrimination for risk stratification.

Furthermore, based on the results summarized in Table 5, models derived from the T1 sequence demonstrated more consistent survival stratification across the three MRI sequences. Clear and stable survival curve separation was observed in both the training and testing sets, further supporting the potential value of the T1-based model for predicting recurrence and metastasis risk in NPC.

Taken together, the CR Model of the GTVp–MLN combination constructed based on MSI features effectively identified patient subgroups with different prognostic risks and demonstrated robust performance in risk stratification and generalization.

## 4. Discussion

In this study, MRI data from 725 NPC patients were used to develop prognostic models based on MSI features, and the predictive performance of different MRI sequences and spatial region combinations was systematically evaluated. The results demonstrated that MSI features effectively capture intratumoral spatial heterogeneity and provide good discriminative ability for prognostic risk prediction. Compared with models derived from a single region, models integrating both the GTVp and MLN regions achieved superior predictive performance. Furthermore, incorporating clinical variables into the imaging model further improved prediction accuracy, highlighting the complementary value of multi-modal information integration in NPC prognostic assessment.

In recent years, the application of radiomics for prognostic prediction in NPC has steadily increased. Previous studies have shown that MRI-based radiomic models can partially predict survival outcomes in NPC patients [32,33]. However, conventional radiomics typically extracts global features from the entire ROI and may not fully capture the complex spatial heterogeneity within tumors. Increasing evidence suggests that intratumoral heterogeneity is closely associated with tumor aggressiveness, treatment response, and metastatic potential. In this study, habitat analysis was used to derive MSI features, which quantify the spatial interactions among different tumor habitats and provide a more comprehensive representation of tumor microenvironment heterogeneity. Compared with conventional whole-tumor radiomics, this strategy offers improved biological interpretability by preserving subregional spatial organization.

Among the evaluated MRI sequences, the T1-based models demonstrated the most favorable and consistent predictive performance. A possible explanation is that T1 sequence may better preserve the structural boundaries and intrinsic tissue contrast required for stable habitat partitioning, thereby enabling more robust characterization of subregional interactions. In contrast, T1C and T2 sequences may be more susceptible to variability introduced by vascular enhancement patterns, edema, and fluid-related signal heterogeneity [34], which could influence habitat clustering stability. These findings suggest that sequence selection plays an important methodological role in habitat-based prognostic modeling and should be carefully considered in future studies.

Another important finding is the superior performance of the combined GTVp-MLN strategy. Most previous radiomics studies have primarily focused on the GTVp, while MLN have received less attention. In NPC, however, MLN is highly prevalent and closely associated with recurrence and distant metastasis risk. By jointly modeling the habitat subregions of both GTVp and MLN, the proposed framework can capture not only intratumoral heterogeneity within each lesion but also the potential spatial interaction patterns between the primary tumor and nodal disease [35]. This combined regional strategy may therefore better reflect the overall tumor burden and metastatic evolutionary behavior, which explains its superior prognostic performance.

From a clinical perspective, the proposed model framework based on habitat analysis may provide practical value for personalized risk stratification in NPC. The ability to distinguish high-risk and low-risk patients using routine pre-treatment MRI could facilitate individualized treatment intensification, follow-up scheduling, and surveillance strategies. In particular, the superior performance of the CR Model suggests that combining quantitative habitat biomarkers with conventional clinical indicators may improve decision support for precision oncology in NPC.

Although larger multi-center cohorts are always desirable, the present study included 725 patients with complete MRI and survival data, which represents a relatively large sample size for a single-center habitat analysis study in NPC, particularly considering the requirement for high-quality MRI data, ROI delineation of both GTVp and MLN regions, and complete survival follow-up. In addition, the consistent superiority of the T1-based and combined GTVp-MLN strategies across multiple comparative analyses further supports the reliability and representativeness of the obtained findings. Although explicit feature selection was not performed, the MSI features were derived from a predefined six-subregion habitat interaction framework rather than unrestricted high-dimensional radiomic extraction, and penalized Cox regression with L2 regularization was further used to improve model robustness and reduce the risk of overfitting.

Despite these findings, several limitations should be acknowledged. First, this was a single-center retrospective study, and multi-center studies with external validation are required to further evaluate the generalizability and robustness of the proposed framework. Second, the present study performed a static analysis using pre-IC imaging, whereas tumor characteristics may dynamically evolve during therapy. The systematic review by Nardone et al. indicates that dynamic imaging features are expected to serve as an important bridge connecting tumor imaging characteristics and prognosis prediction [36]. In addition, the multi-parameter MRI technique, by integrating the complementary information provided by different sequences, is expected to more comprehensively reflect the biological heterogeneity of tumors [37,38]. Future studies incorporating temporal imaging information, longitudinal habitat evolution, and multi-parametric MRI fusion strategies may further improve the predictive performance and clinical applicability of R Models based on habitat analysis.

## 5. Conclusions

In this study, we developed and evaluated the R Model and CR Model based on MSI features derived from habitat analysis. First, among the T1, T1C, and T2 sequences, the T1-based models demonstrated the most favorable prognostic performance, highlighting the importance of MRI sequence selection in habitat analysis. Second, the incorporation of habitat subregional information from MLN regions provided additional prognostic value beyond the GTVp alone. Third, among the evaluated regional strategies, the combined GTVp-MLN model achieved the best overall predictive performance, indicating that joint characterization can better capture tumor heterogeneity. Furthermore, incorporating clinical variables into the CR Model further improved prediction accuracy, and the T1-based CR Model using the combined GTVp-MLN region showed the highest predictive performance.

The main strength of this study lies in the comparative evaluation of multiple MRI sequences, regional combinations, and both the R Model and CR Model within a relatively large single-center cohort. However, the retrospective single-center design and the use of only static pre-IC MRI data remain as limitations. Future studies should focus on multi-center validation, dynamic habitat evolution, and multi-parametric MRI integration to further improve the robustness and clinical applicability of the proposed models.

## Figures and Tables

**Table 1:** Summary of 58 MSI features.

Feature Names	Feature Description
MSI 1–MSI 4	Second-order statistical features: Contrast, homogeneity, correlation, energy of GLCM
MSI 5–MSI 10	Absolute volume of tumor subregions
MSI 11–MSI 16	Absolute interaction between tumor subregions and boundaries
MSI 17–MSI 21	Absolute interaction between tumor subregion 1 and other subregions
MSI 22–MSI 25	Absolute interaction between tumor subregion 2 and subregions 3, 4, 5, 6
MSI 26–MSI 28	Absolute interaction between tumor subregion 3 and subregions 4, 5, 6
MSI 29–MSI 30	Absolute interaction between tumor subregion 4 and subregions 5, 6
MSI 31	Absolute interaction between tumor subregion 5 and subregions 6
MSI 32–MSI 37	Volume proportion of tumor subregions
MSI 38–MSI 43	Normalized interaction between tumor subregions and boundaries
MSI 44–MSI 48	Normalized interaction between tumor subregion 1 and other subregions
MSI 49–MSI 52	Normalized interaction between tumor subregion 2 and subregions 3, 4, 5, 6
MSI 53–MSI 55	Normalized interaction between tumor subregion 3 and subregions 4, 5, 6
MSI 56–MSI 57	Normalized interaction between tumor subregion 4 and subregions 5, 6
MSI 58	Normalized interaction between tumor subregion 5 and subregions 6

**Table 2:** Characteristics of patients in training and testing set.

Characteristic	All	Training (n = 543)	Testing (n = 182)	*p*-Value
Median age, years, no. (%)				
<43	349 (48.1)	259 (47.7)	90 (49.5)	0.7461
≥43	376 (51.9)	284 (52.3)	92 (50.5)	
Gender, no. (%)				
Male	552 (76.1)	415 (76.4)	137 (75.3)	0.8296
Female	173 (23.9)	128 (23.6)	45 (24.7)	
WHO pathological type, no. (%)				
Type I–II	13 (1.8)	8 (1.5)	5 (2.7)	0.4248
Type III	712 (98.2)	535 (98.5)	177 (97.3)	
T stage, no. (%)				
T1	47 (6.5)	38 (7.0)	9 (4.9)	0.7156
T2	60 (8.3)	44 (8.1)	16 (8.8)	
T3	398 (54.9)	300 (55.2)	98 (53.8)	
T4	220 (30.3)	161 (29.7)	59 (32.4)	
N stage, no. (%)				
N0	20 (2.8)	15 (2.8)	5 (2.7)	0.8995
N1	258 (35.6)	189 (34.8)	69 (37.9)	
N2	251 (34.6)	190 (35.0)	61 (33.5)	
N3	196 (27.0)	149 (27.4)	47 (25.8)	
Overall stage, no. (%)				
III	355 (49.0)	268 (49.4)	87 (47.8)	0.7817
IVA	370 (51.0)	275 (50.6)	95 (52.2)	
Pre-IC EBV-DNA (copies/mL), no. (%)				
<4000	300 (41.4)	224 (41.3)	76 (41.8)	0.9737
≥4000	425 (58.6)	319 (58.7)	106 (58.2)	
CCRT, no. (%)				
No	60 (8.3)	47 (8.7)	13 (7.1)	0.6272
Yes	665 (91.7)	496 (91.3)	169 (92.9)	

**Table 3:** Quantitative comparison of different habitat subregion numbers for determining the optimal clustering scheme.

Number of Habitats (*k*)	Silhouette Coefficient	CH Score	MSI Feature Dimension
4	0.43	196	32
6	**0.61**	**238**	58
8	0.52	219	92

*Note:* Bold values indicate the optimal clustering number (*k* = 6) with the highest Silhouette Coefficient and CH Score.

**Table 4:** Prognostic performance of **R Model** based on different MRI sequences and tumor regions.

		T1		T1C		T2	
		Training	Testing	Training	Testing	Training	Testing
GTVp	C-Index	0.689	0.652	0.666	0.664	0.652	0.641
	(95% CI)	(0.618–0.747)	(0.541–0.758)	(0.609–0.724)	(0.562–0.759)	(0.590–0.713)	(0.533–0.747)
	*p*-Value	<0.001	0.02	<0.001	0.009	<0.001	0.090
	AUC	0.706	0.661	0.670	0.665	0.678	0.659
MLN	C-Index	0.659	0.644	0.629	0.620	0.634	0.628
	(95% CI)	(0.599–0.715)	(0.536–0.743)	(0.560–0.694)	(0.512–0.723)	(0.573–0.693)	(0.512–0.748)
	*p*-Value	0.005	0.072	0.120	0.068	0.041	0.010
	AUC	0.676	0.658	0.639	0.623	0.654	0.634
GTVp-MLN	C-Index	**0.713**	**0.693**	0.709	0.684	0.688	0.674
	(95% CI)	(0.661–0.764)	(0.607–0.776)	(0.655–0.761)	(0.597–0.763)	(0.630–0.743)	(0.557–0.774)
	*p*-Value	<0.001	<0.001	<0.001	0.061	<0.001	<0.001
	AUC	**0.724**	**0.712**	0.721	0.686	0.712	0.693

*Note:* Bold values represent the best prognostic performance (highest C-Index and AUC) among all models and sequences.

**Table 5:** Prognostic performance of **CR Model** based on different MRI sequences and tumor regions.

		T1		T1C		T2	
		Training	Testing	Training	Testing	Training	Testing
GTVp	C-Index	0.717	0.704	0.706	0.692	0.704	0.677
	(95% CI)	(0.659–0.770)	(0.612–0.791)	(0.649–0.765)	(0.583–0.787)	(0.640–0.765)	(0.579–0.779)
	*p*-Value	<0.001	0.020	<0.001	<0.001	<0.001	<0.001
	AUC	0.744	0.723	0.715	0.698	0.726	0.685
MLN	C-Index	0.683	0.660	0.653	0.641	0.668	0.650
	(95% CI)	(0.625–0.741)	(0.547–0.761)	(0.589–0.718)	(0.532–0.742)	(0.604–0.727)	(0.536–0.750)
	*p*-Value	<0.001	0.001	0.005	0.004	0.052	0.008
	AUC	0.699	0.680	0.660	0.646	0.684	0.664
GTVp-MLN	C-Index	**0.735**	**0.722**	0.726	0.719	0.716	0.711
	(95% CI)	(0.670–0.789)	(0.619–0.812)	(0.676–0.775)	(0.613–0.805)	(0.657–0.772)	(0.616–0.789)
	*p*-Value	<0.001	<0.001	<0.001	<0.001	<0.001	<0.001
	AUC	**0.756**	**0.742**	0.742	0.723	0.739	0.723

*Note:* Bold values represent the best prognostic performance (highest C-Index and AUC) among all models and sequences.

## Data Availability

The MRI data used in this study were provided by Sun Yat-sen University Cancer Center are not publicly available due to patient privacy and institutional restrictions, but may be made available from the corresponding author upon reasonable request and with permission from the hospital ethics committee. Additional code and data from our research can be obtained by reaching out to the corresponding author.

## Appendix A. Treatment

All patients received radical IMRT. Target volume delineation was performed in accordance with International Commission on Radiological Units and Measurements (ICRU) Reports 50, 62, and 83. The target volumes for NPC included the gross tumor volume (GTV), clinical target volume (CTV), and planning target volume (PTV). The GTV encompassed visible tumor on clinical or imaging examination, subdivided into the GTVp and the nodal gross tumor volume (GTVn). The CTV encompassed potential subclinical disease around the GTV and was stratified into high risk (CTV1) and low risk (CTV2) regions. A 3-mm margin was added to the GTV (generating PGTVp and PGTVn) and CTV (generating PCTV1 and PCTV2) to create the PTV, accounting for organ motion, system errors, and setup uncertainty. The prescribed radiation doses were: PGTVp, 66–72 Gy in 28–33 fractions; PGTVn, 64–70 Gy in 28–33 fractions; PCTV1, 60–63 Gy in 28–33 fractions; and PCTV2, 54–56 Gy in 28–33 fractions.

All patients completed 2–4 cycles of IC prior to radiotherapy: 46.9% (340/725) received 2 cycles, 44.3% (321/725) received 3 cycles, and the remainder completed 4 cycles. During radiotherapy, 91.7% (665/725) of patients received concurrent chemoradiotherapy (CCRT).

IC regimens included three-week cycles of TPF (docetaxel, cisplatin, and fluorouracil), TP (docetaxel and cisplatin), or PF (cisplatin and fluorouracil). CCRT consisted primarily of platinum-based chemotherapy administered either every three weeks (80–100 mg/m^2^ for 2–3 cycles) or weekly (30–40 mg/m^2^ for 5–6 cycles), typically starting on the first day of IMRT.

## Appendix B. MRI Acquisition and Preprocessing Protocol

All patients underwent magnetic resonance imaging (MRI) scans. The MRI equipment used includes 1.0T (Philips Panorama HFO), 1.5T (GE SIGNA EXCITE, GE Signal HDxt, SIEMENS Espree) and 3.0T (GE DISCOVERY MR750, GE Model scanners: DISCOVERY MR750w, PHILIPS Achieva, SIEMENS TrioTim. Three MRI sequences were collected for each patient: T1, T2, and T1C.

The MRI scan parameters are as follows: the layer thickness is 3–6 mm, and the layer spacing is 0.3–1.65 mm. The repeat time (TR) of the T1 sequence is 597.8 ms, ranging from 400.0 to 984.0 ms, and the echo time (TE) is 8.6 ms, ranging from 7.0 to 15.0 ms. The TR of the T1C sequence is 597.8 ms, with a range of 333.0–1060.0 ms, and the TE is 8.7 ms, with a range of 7.0–16.0 ms. The TR of the T2 sequence is 3655.5 ms, ranging from 2000.0 to 8810.0 ms, and the TE is 87.4 ms, ranging from 77 to 129 ms. The image matrix ranges from 256 × 256 to 768 × 768, the field of view ranges from 248 × 180 to 512 × 320 mm, and the planar spatial resolution ranges from 0.29 × 0.29 to 0.94 × 0.94 mm.

In this study, the T1, T2 and T1C sequence MRI images of the patients were extracted from the hospital PACS system (GE Healthcare Centricity RIS CE and Carestream, Ontario, Canada), and the initial format of the images was DICOM. Due to the differences in the models of scanning devices and magnetic field intensities, there is a certain degree of inconsistency in the gray scale of the images. Therefore, the DICOM format is first converted to the NIfTI format, and the SimpleITK tool is used to perform N4 bias field correction on the MRI images to eliminate non-parametric and non-uniform intensity deviations, thereby achieving the standardization of the gray-scale distribution. After correction, the gray-scale distribution of different sequence images becomes more consistent. In addition, considering that the layer thickness and pixel resolution of images from different patients vary, this study adopts a unified discretization method to discretize the image intensity into 64 gray levels, and resampling the voxels to isotropic 2 × 2 × 2 cm^3^ through linear interpolation to enhance the consistency and accuracy of spatial analysis.
